# Characterization and pathogenicity of a novel avian orthoreovirus in China

**DOI:** 10.3389/fmicb.2024.1529351

**Published:** 2025-01-09

**Authors:** Shunyan Chen, Jialin Yang, Li Li, Yawei Guo, Shenghua Yang, Zetao Su, Sucan Zhao, Xuesong Li, Wencheng Lin, Yunping Du, Lijuan Yin, Lianxiang Wang, Feng Chen

**Affiliations:** ^1^College of Animal Science, South China Agricultural University, Guangzhou, China; ^2^Yunfu Branch, Guangdong Laboratory for Lingnan Modern Agriculture, Yunfu, China

**Keywords:** avian orthoreovirus, isolation, genomic analysis, phylogenetic analysis, pathogenicity

## Abstract

**Introduction:**

Avian orthoreovirus (ARV) is a significant pathogen causing viral arthritis, leading to substantial economic losses in the poultry industry worldwide.

**Methods:**

A novel ARV strain, designated FJ202311, was isolated from a broiler farm in Fujian Province, China. Whole-genome sequencing was conducted using next-generation sequencing with MGI technology, and phylogenetic analysis of the sigma C amino acid sequence was performed. Comparative sequence analysis identified unique genetic features of FJ202311. Pathogenicity studies were carried out by inoculating broilers with the isolated strain and monitoring clinical signs, weight gain, and histopathological changes.

**Results:**

The complete genome of FJ202311 was determined to be 23,495 base pairs in length, encoding 12 major proteins. Phylogenetic analysis revealed that FJ202311 forms a distinct genotypic cluster, exhibiting only 47.1% to 59.3% sequence identity to 16 reference ARV strains. Notably, 50 unique amino acid substitutions were identified in the sigma C protein. Pathogenicity tests demonstrated that FJ202311 caused severe arthritis and tenosynovitis in broilers. Infected birds exhibited significant weight loss compared to controls, with reductions of 11.78% and 8.93% at 14 and 21 days post-infection, respectively.

**Discussion:**

This study highlights the unique molecular and pathogenic characteristics of the novel ARV strain FJ202311, contributing to our understanding of ARV diversity and epidemiology in China. These findings underscore the importance of continuous monitoring and provide insights for developing improved prevention and control strategies against ARV.

## Introduction

Avian orthoreovirus (ARV) is a significant avian pathogen associated with various diseases in chickens, leading to substantial economic losses in the global poultry industry. ARV-induced viral arthritis and tenosynovitis primarily affect the joints and tendons, causing swelling, lameness, and reduced mobility, which negatively impact feed efficiency and weight gain (Sellers, 2017). Hepatitis, characterized by liver inflammation and dysfunction, can result in systemic effects such as jaundice and impaired growth (Mandelli et al., 1978). Respiratory diseases manifest as coughing, sneezing, and labored breathing, often exacerbated by secondary bacterial or viral infections (Fahey and Crawley, 1954). Runting-stunting syndrome is marked by uneven growth and underdeveloped chicks, leading to economic losses due to poor flock uniformity (Goodwin et al., 1993). Malabsorption syndrome causes nutrient malabsorption, diarrhea, poor growth, and reduced performance (Page et al., 1982). Neurological signs, including tremors, ataxia, and paralysis, significantly impair bird welfare (Van de Zande and Kuhn, 2007). Immunosuppression compromises the immune system, increasing susceptibility to secondary infections and reducing vaccine efficacy (Neelima et al., 2003). ARV infects various avian species, including chickens (Chen et al., 2019), turkeys (Sharafeldin et al., 2014), ducks (Wang et al., 2020), geese (Palya et al., 2003), pigeons (Vindevogel et al., 1982), ostriches (Sakai et al., 2009), and wild birds (Huhtamo et al., 2007). The virus is transmitted both horizontally and vertically, with intestinal shedding lasting longer than respiratory shedding, making contaminated feces a primary source of contact transmission. Vertical transmission occurs through eggs, allowing infected breeder hens to pass the virus to a small proportion of chicks (Sellers, 2022; Rafique et al., 2024). The first ARV strain was isolated in 1954 from chickens with respiratory disease (Fahey and Crawley, 1954), and its role as a causative agent of viral arthritis was identified in Walker et al. (1972). ARV infections have since spread globally, causing economic losses in regions including the USA (Lu et al., 2015), Israel (Dawe et al., 2022), Brazil (De Carli et al., 2020), Canada (Palomino-Tapia et al., 2018), Korea (Noh et al., 2018), and China (Teng et al., 2014).

ARV is a member of the genus *Orthoorthoreovirus* of the family *Reoviridae,* and has double-stranded RNA genome with 10 segments (Rafique et al., 2024). Based on their electrophoretic mobility, ARV genome can be classified into three size classes: three large segments (L1, L2, L3), three middle segments (M1, M2, M3) and four small segments (S1, S2, S3, S4) (Rafique et al., 2024), encoding 8 structural proteins (λA, λB, λC, μA, μB, σA, σB, and σC) and 4 non-structural proteins (μNS, σNS, p10, and p17) (Varela and Benavente, 1994). The λA protein, encoded by the L1 gene, plays a crucial role in viral replication and assembly. The λB protein, encoded by the L2 gene, facilitates RNA polymerase activity, which is essential for viral RNA synthesis. The λC protein, encoded by the L3 gene, forms turret-like structures at the fivefold axis of the core, enabling interactions with the outer capsid during viral assembly. The μA protein, encoded by the M1 gene, contributes to the stability and integrity of the capsid. The μB protein, encoded by the M2 gene, undergoes myristoylation, which is necessary for its functionality, aiding viral replication and the formation of viral factories. The μNS protein, encoded by the M3 gene, accumulates in viral factories, potentially assisting in RNA packaging and replication. The σA protein, encoded by the S2 gene, interferes with host antiviral responses by inhibiting dsRNA-dependent protein kinases and IFN production. The σB protein, encoded by the S3 gene, is essential for pathogenesis and the elicitation of group-specific neutralizing antibodies. The σNS protein, encoded by the S4 gene, accelerates RNA folding and promotes specific RNA–RNA interactions required for genome replication. The p10 protein, encoded by the S1 gene, enhances membrane permeability, induces syncytium formation, and triggers apoptosis, contributing to virulence. The p17 protein, also encoded by the S1 gene, accumulates in the nucleus to modulate immune responses, block signaling pathways, and regulate interferon production. Among these proteins, the sigma C (σC) protein, encoded by the S1 segment, is highly variable, involving viral attachment and entry and the production of type-specific neutralizing antibodies (Liu et al., 2003; Rafique et al., 2024). Accrording to the amino acid sequence of the σC protein, ARV isolates are usually classified into six genotypes: I, II, III, IV, V, and VI (Tang et al., 2016; De la Torre et al., 2021).

In China, ARV infection was first reported in 1985 (Chen et al., 2019). Although several commercial vaccines against ARV are available, the cases of viral arthritis induced by ARV variants increased in Chinese poultry farms in recent years, indicating that these commercial vaccines might confer partial protection against different novel ARV strains (Chen et al., 2019; Yan et al., 2021; Jiang et al., 2022). While several ARV strains have been identified and studied, there is limited information on the genetic diversity and pathogenicity of emerging ARV variants circulating in China. Additionally, the role of the highly variable S1 gene in ARV virulence and immune evasion remains poorly understood. In this study, we isolated a novel ARV strain from a chicken flock in Fujian province of China. The whole-genome sequencing and analysis revealed that this strain is genetically distinct from known ARV strains, particularly exhibiting extremely significant variability in the S1 gene compared to other genotypic clusters. Furthermore, pathogenicity analysis indicated that this novel strain causes severe arthritis and tenosynovitis in broiler chickens. These findings enhanced our understanding of the epidemiological evolution of ARV and provided essential insights for the control of ARV.

## Materials and methods

### Sample collection and processing

In November 2023, the clinical samples (including tendon and cecal tonsils) were collected from 25-day-old white-feathered Cobb broilers exhibiting arthritis, tenosynovitis, and poor production performance in Fujian Province. These samples were homogenized in phosphate-buffered saline (PBS) at pH 7.4 to create a 20% (w/v) tissue suspension. The suspensions underwent 3 cycles of freezing and thawing, followed by clarification through centrifugation at 6,000 × g for 5 min at 4°C. The supernatants were subsequently harvested for virus isolation and viral nucleic acid extraction. Viral nucleic acid extraction was performed using the MagaBio Plus Viral DNA/RNA Purification Kit (Bioer, Hangzhou, China) and the nucleic acid purification system (Bioer, Hangzhou, China), following the manufacturer’s protocol.

### Detection of ARV and other pathogens

The M1 gene of ARV was amplified using universal primers and probe (Table 1) by the real-time polymerase chain reaction (PCR) assay as previously described (Yan et al., 2021). To determining co-infection, the existences of other pathogens, including *Mycoplasma synoviae* (MS) (Huang et al., 2015), fowl adenovirus (FAdV) (Li et al., 2020), chicken infectious anemia virus (CIAV) (Li et al., 2020), avian influenza virus (AIV) (Bo et al., 2021), infectious bronchitis virus (IBV) (Laamiri et al., 2018), and Newcastle disease virus (NDV) (Zhang et al., 2020), were detected from ARV-positive samples by real-time PCR with primers and probes (Table 1) as described previously with minor modifications.

**Table 1:** Primers and probes used in this study.

Pathogen	Primer/probe	Nucleotide sequence (5′-3′)	Target gene	Product (bp)	Reference
MS	Forward	ATAGCAATTTCATGTGGTGATCAA	vlhA	143	Huang et al. (2015)
	Reverse	TGGATTTGGGTTTTGAGGATTA			
	Probe	ROX-CAGCACCTGAACCAACACCTGGAA-Eclipse			
IBV	Forward	GCTTTTGAGCCTAGCGTT	5’-UTR	143	Laamiri et al. (2018)
	Reverse	GCCATGTTGTCACTGTCTATTG			
	Probe	FAM-CACCACCAGAACCTGTCACCTC-BHQ1			
AIV	Forward	AGGGTTTGTGTTCACGCTC	M	186	Bo et al. (2021)
	Reverse	CCGGTTGAGTAGCTGAGTGC			
	Probe	ROX-CCGTGCCCAGTGAGCGAGGAC-BHQ1			
CIAV	Forward	ATCAACCCAAGCCTCCCT	VP2	145	Li et al. (2020)
	Reverse	CTCGTCTTGCCATCTTACAG			
	Probe	Cy5-TACCACTACTCCCAGCCGACCCC-BHQ2			
FAdV	Forward	AAAACTGAGACTTTCCCACAA	ORF14	162	Li et al. (2020)
	Reverse	AGATACCCTCCGAAGAACTAC			
	Probe	HEX-TCTCCCATATCATTTCCATGCCTCC-BHQ1			
NDV	Forward	GACTCAACTCTTGGGCATACA	F	172	Zhang et al. (2020)
	Reverse	TGAGGTGTCAAGCTCTTCTAT			
	Probe	FAM-CAGTCGGGAACCTAAATAATATGCGTGC-BHQ1			
ARV	Forward	ATGGCCTATCTAGCCACACCTG	M1	89	Yan et al. (2021)
	Reverse	CAACGTGATAGCATCAATAGTAC			
	Probe	FAM-TGCTAGGAGTCGGTTCTCGCA-BHQ1			
	Forward	GCTTTTTCTCCGAACGCCGAAATG	L1	3,959	In this study
	Reverse	GATGAATAATCTCCAACGAG			
	Forward	GCTTTTTCCTCACCATGCATGTCA	L2	3,830	
	Reverse	GATGAGTAATTCCTCGAGCCA			
	Forward	GCTTTTTCACCCATGGCTCAGATTA	L3	3,907	
	Reverse	GATGAGTAACACCCTTCTACT			
	Forward	GCTTTTCTCGACATGGCCTATCTAG	M1	2,283	
	Reverse	GATGAGTATCTCAAGACGAC			
	Forward	GCTTTTTCAGTGCCAATCTTTCTCA	M2	2,158	
	Reverse	GATGAATAACGTGCCAATCC			
	Forward	GCTTTTTGAGTCCTAGCGTGGATCATG	M3	1996	
	Reverse	GATGAATAACCGAGTCCGCCG			
	Forward	GCTTTTTCAGTCCTTCGTGTCAATGTT	S1	1,643	
	Reverse	GATGAATAACCAGTCCCCTTA			
	Forward	GCTTTTTCTCCCACGATGGCGCGTG	S2	1,324	
	Reverse	GATGAGTACACCCACGTGCTG			
	Forward	GCTTTTTGAGTCCTTAGCGT	S3	1,202	
	Reverse	GATGAATAGGCGAGTCCCGCTA			
	Forward	GCTTTTTGAGTCCTTGTGCA	S4	1,192	
	Reverse	GATGAATAAGAGTCCAAGTCAC			

For four pathogens (ARV, AIV, IBV, NDV), the extracted viral RNA was tested by real-time PCR by using the Hifair V C58P2 Multiplex One Step RT-qPCR Probe Kit (Yeasen, Shanghai, China) under the following reaction conditions: 50°C for 20 min; 95°C for 5 min; the cycling step was repeated for 40 cycles at 95°C for 15 s, 60°C for 30 s. For three other pathogens (MS, FAdV and CIAV), the extracted DNA was tested by real-time PCR by using the Hieff Unicon^®^ Universal TaqMan multiplex qPCR master mix (Yeasen, Shanghai, China) under the following reaction conditions: 95°C for 5 min; the cycling step was repeated for 40 cycles at 95°C for 15 s, 60°C for 30 s.

### Virus isolation in LMH cells

For virus isolation, only ARV-positive tissue samples were used to isolate ARV on Leghorn male hepatoma (LMH) cells (ATCC #CRL-2117). Briefly, the LMH cells were maintained in Dulbecco’s modified Eagle’s medium (DMEM) (Hyclone, Logan, UT, USA) supplemented with 10% fetal bovine serum (FBS) (BOVOGEN, Melbourne, Australia) and 1% antibiotic-antimycotic (Gibco, Grand Island, NY, USA) at 37°C in a 5% CO_2_ incubator. The ARV-positive supernatant was filtered through a 0.22 μm filter (Millipore, Tullagreen, Carrigtwohill, Ireland). The sterile filtrates were then overlaid onto LMH cell monolayers in 12-well plates. After 1 h of absorption at 37°C, the supernatant was discarded, the LMH cells were washed three times with PBS, and fresh DMEM supplemented with 2% FBS and 1% antibiotic-antimycotic was added. After a 72-h incubation period, the supernatant and cells were harvested through three freeze–thaw cycles for the next round of virus propagation and real-time PCR detection. Following three blind passages of infected cells, the culture supernatants were harvested and stored at −80°C for further analysis. Additionally, the median tissue culture infectious dose (TCID_50_) was determined as previously described (Yan et al., 2021).

### Electron microscopy

Electron microscopic observation was conducted as described previously with some modifications (De Carlo and Harris, 2011). Briefly, ARV-infected LMH cells were harvested and subjected to three freeze–thaw cycles, and centrifugated at 8,000× g for 30 min at 4°C. The mixture was then ultracentrifuged at 100,000 × g for 2 h at 4°C. The pellet was resuspended in PBS buffer and negatively stained with 2% phosphotungstic acid. After blotting and drying, the grids were examined using an electron microscope (Leica, Wetzlar, Germany).

### Immunofluorescence assay (IFA)

The immunofluorescence assay (IFA) was performed with modifications as described previously (Yan et al., 2021). Briefly, ARV-infected or mock-infected LMH cell monolayers were washed twice with PBS and fixed with 4% paraformaldehyde for 30 min at room temperature. The fixed cells were then permeabilized with 0.5% Triton X-100 (Solarbio, Beijing, China) for 30 min, blocked with 3% bovine serum albumin (BSA) for 1 h at room temperature, and washed three times with PBS. Subsequently, the cells were incubated with an anti-ARV p10 antibody (Zhongnong Yiyou Medical Biotechnology Co., Ltd., Guangdong, China) at a dilution of 1:500 overnight at 4°C. After three washes with PBS, the cells were incubated with FITC-conjugated goat anti-mouse IgG secondary antibody (Beyotime, Shanghai, China) at a dilution of 1:1,000 for 1 h at at room temperature. All images were captured and processed using a fluorescence microscope (Leica, Wetzlarm, Germany).

### High-throughput sequencing and analysis

Total RNA was extracted from the FJ202311 strain using a Magnetic Bead Method Nucleic Acid Extraction Kit (Baybiopure, Guangzhou, China) according to the manufacturer’s protocol. Following RNA extraction, ribosomal RNA (rRNA) was removed using the MGIEasy rRNA Depletion Kit (MGI Tech). RNA libraries were then prepared using the MGISP-Smart 8 sample preparation system (MGI Tech). Finally, the RNA libraries were sequenced on an MGISEQ-2000RS sequencer (MGI Tech) using 100-nt single-read sequencing.

The CLC Genomics Workbench version 24.0.1 (Qiagen) was used for viral genome assembly. Adapters and non-target sequences, including mRNA, rRNA, and chicken sequences, were trimmed off. Sequence assembly and analysis were performed using the *de novo* assembly tool and the map reads to reference tool within the CLC software, employing default parameters as previously described (Zeden and Grundling, 2023).

Additionally, ten PCR primers were designed (Table 1), and PCR amplification followed by Sanger sequencing was performed to confirm the results of next-generation sequencing. Briefly, all gel-purified PCR products were cloned into the pMD19-T vector (TaKaRa, Dalian, China) and then transformed into *E. coli* DH5α competent cells (TaKaRa, Dalian, China). Three or more positive clones for each PCR product were selected and sent to Sangon Biotech Company (Guangzhou, China) for sequencing. The nucleotide sequences were submitted to GenBank.

### Sequence comparison, phylogenetic analysis and recombination analysis

The homology identity of nucleotide and amino acid sequences was determined using 16 ARV reference sequences with the EditSeq and MegAlign programs of the DNAstar Lasergene 7.1 software (DNAStar, Madison, WI, USA). Sequence alignments were performed using the Clustal W method, and phylogenetic analysis of the nucleotide and amino acid sequences was constructed using the neighbor-joining method with 1,000 bootstrap replicates in the MEGA software (version 7.0). Comparative analysis of whole genome alignment of the FJ202311 strain and the 16 ARV reference strains (Table 2) was conducted on the mVISTA online platform.^1^

**Table 2:** The 16 ARV reference strains and the FJ202311 strain retrieved from the GenBank database used in the analysis.

No.	Strain	σC based genotype	GenBank number									
			L1	L2	L3	M1	M2	M3	S1	S2	S3	S4
1	MS01	1	KY860642	KY860641	KY860640	KY860639	KY860638	KY860637	KY860636	KY860635	KY860634	KY860633
2	526	1	KF741696	KF741697	KF741698	KF741699	KF741700	KF741701	KF741702	KF741703	KF741704	KF741705
3	1733	1	KF741706	KF741707	KF741708	KF741709	KF741710	KF741711	KF741712	KF741713	KF741714	KF741715
4	C78	1	KF741716	KF741717	KF741718	KF741719	KF741720	KF741721	KF741722	KF741723	KF741724	KF741725
5	S1133	1	KF741756	KF741757	KF741758	KF741759	KF741760	KF741761	KF741762	KF741763	KF741764	KF741765
6	T-98	1	EU616739	JN641889	EU616738	EU616736	EU616742	EU616743	EF057398	JN641887	EF030499	JN641884
7	PHC-2020-0545	2	MW174784	MW174785	MW174786	MW174787	MW174788	MW174789	MW174790	MW174791	MW174792	MW174793
8	Reo-PA-Turkey-22,342–13	2	KP173683	KP173684	KP173685	KP173686	KP173687	KP173688	KP173689	KP173690	KP173691	KP173692
9	D1007	2	KR476798	KR476800	KR476799	KR476801	KR476802	KR476803	KR476804	KR476805	KR476806	KR476807
10	AHZJ19	3	OK077993	OK077994	OK077995	OK077996	OK077997	OK077998	OK077999	OK078002	OK078003	OK078004
11	Reo-PA-Layer-01224A-14	3	KT428298	KT428299	KT428300	KT428301	KT428302	KT428303	KT428304	KT428305	KT428306	KT428307
12	K1600657	4	MK583331	MK583332	MK583333	MK583334	MK583335	MK583336	MK583337	MK583338	MK583339	MK583340
13	SDYT2020	4	MW394456	MW394457	MW394458	MW394459	MW394460	MW394461	MW394462	MW394463	MW394464	MW394465
14	LY383	5	MF183221	MF183212	MF183213	MF183214	MF183215	MF183216	MF183217	MF183218	MF183219	MF183220
15	SD26	5	MW244842	MW244843	MW244844	MW244845	MW244846	MW244847	MW244848	MW244849	MW244850	MW244851
16	3,211-V-02	6	KX398272	KX398273	KX398274	KX398275	KX398276	KX398277	KX398278	KX398279	KX398280	KX398281
17	FJ202311		PQ106581	PQ106582	PQ106583	PQ106584	PQ106585	PQ106586	PQ106587	PQ106588	PQ106589	PQ106590

### Pathogenicity analyses of the ARV

To assess the pathogenicity of the ARV isolate FJ202311, challenge studies were performed in chickens. A total of 40 healthy Cobb broilers (7-day-old), confirmed to be free of common avian pathogens, were obtained from Guannan Wen’s Food Co., Ltd., Jiangsu, China. The broilers were randomly assigned to two groups (*n* = 20 per group): an infection group and a control group. The infection group was inoculated via footpad injection with 0.1 mL of virus suspension containing 10^6 TCID_50_ of ARV, while the control group received 0.1 mL of PBS via the same route. Each group was housed separately in isolators under controlled conditions. Clinical signs, body weight, and mortality were recorded throughout the experimental period of 21 days. At 7, 14, and 21 days post-infection (dpi), three chickens from each group were randomly selected and euthanized for necropsy. Tissue samples, including heart, liver, spleen, lung, kidney, bursa of Fabricius, pancreas, small intestine, proventriculus, gizzard, thymus, cecal tonsil, and tendon, along with blood sample and cloacal swab, were collected to evaluate viral distribution and shedding. Additionally, tendon samples were fixed in 10% neutral-buffered formalin for subsequent histopathological analysis. Serum samples were collected from chickens to detect ARV-specific antibodies using a commercial ID Screen^®^ Avian Reovirus indirect enzyme-linked immunosorbent assay (ELISA) kit (IDvet, Grabels, France).

### Statistical analysis

All experiments were conducted independently in triplicate, producing consistent results. Data were expressed as mean ± standard deviations (SD). Statistical significance was evaluated using GraphPad Prism software (version 6.0). A *p*-value of <0.05 was considered statistically significant, while *p*-values of <0.01 were regarded as highly significant.

## Results

### Clinical history and virus detection

The primary clinical symptoms observed in the affected 25-day-old white-feathered Cobb chickens included arthritis, tenosynovitis, and reduced production performance. Post-mortem examinations revealed significant swelling and hemorrhages in the tarsal joints. Out of 30 tissue samples, 19 (63%) tested positive for avian reovirus (ARV) using real-time RT-PCR. All samples were negative for MS, FAdV, CIAV, AIV, IBV, and NDV as confirmed by real-time PCR or real-time RT-PCR.

### Virus isolation and identification

**Figure 1:**
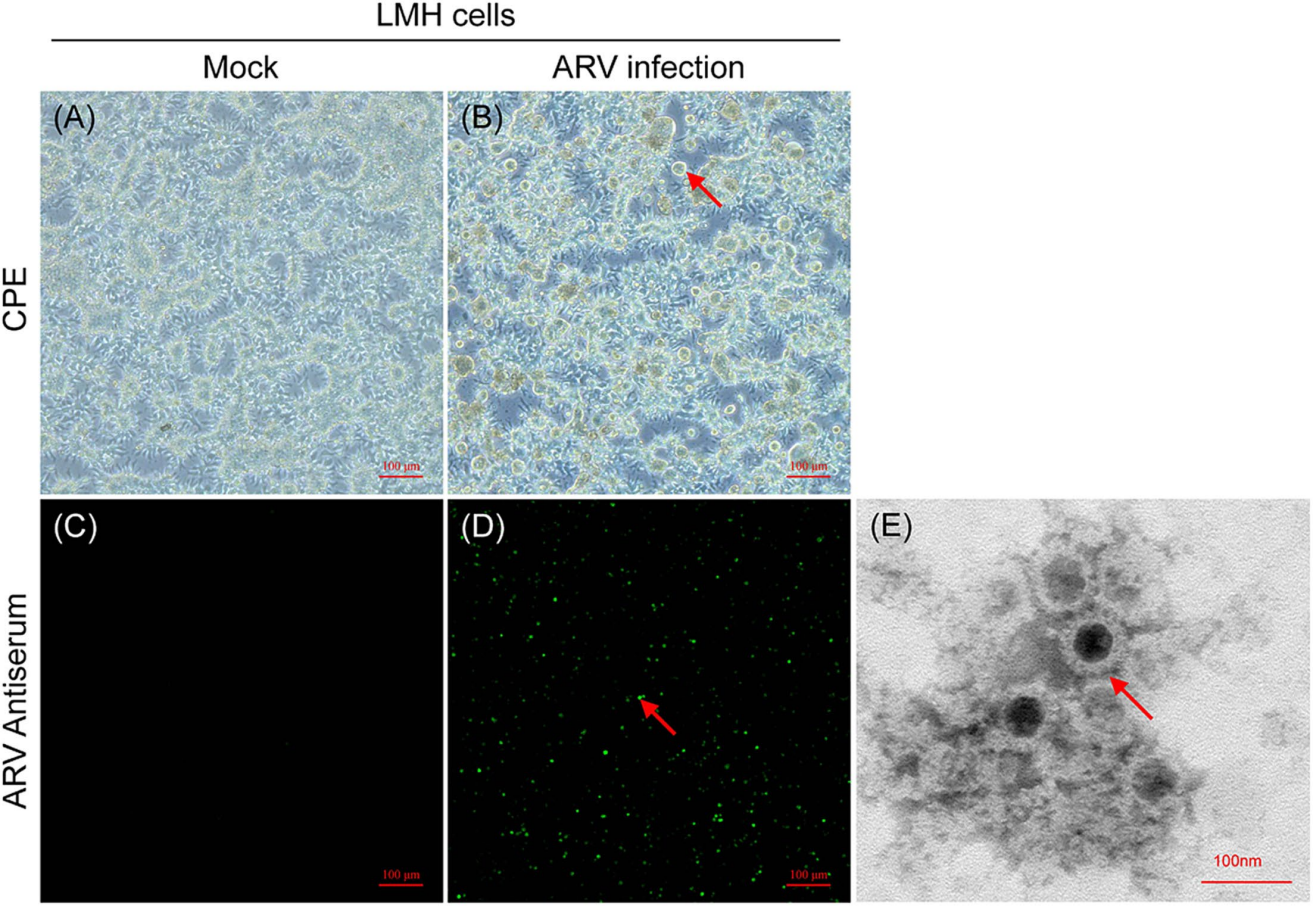
Isolation and characterization of the ARV FJ202311 strain. **(A)** Mock-infected LMH cells at 48 h post-infection (hpi). **(B)** ARV-infected LMH cells showing extensive cell fusion into disc-like structures (indicated by arrows) at 48 hpi. **(C,D)** Immunofluorescence assay (IFA) at 48 hpi using anti-ARV p10 monoclonal antibody (mAb) and FITC-conjugated goat anti-mouse secondary antibody. Specific green fluorescence is observed in infected LMH cells (indicated by arrows). **(E)** Electron micrographs of ARV-inoculated LMH cells. Spherical ARV particles are visible (indicated by arrow).

The ARV strain FJ202311 was isolated on LMH cells through successive generations of culture, and its biological characteristics were evaluated. No cytopathic effect (CPE) were observed in the untreated control LMH cells (Figure 1A). As shown in Figure 1B, the infected LMH cells showed clear cytopathic effect (CPE) after three serial cell passages, and typical syncytial lesions characterized by numerous cells fusing into discs of varying sizes were observed at 48 h post-infection (hpi). To confirm ARV replication in LMH cells, viral RNA was extracted from the infected cells and tested by ARV-specific real-time RT-PCR. The FJ202311 strain was only positive for ARV whereas negative for other common viruses, such as AIV, IBV, NDV, MS, FAdV, and CIAV. No green fluorescence signal was detected in the untreated control LMH cells (Figure 1C). The presence of the ARV strain in LMH cells was further confirmed by immunofluorescence assay (IFA), which detected green fluorescence signals in the LMH cells infected with FJ202311 (Figure 1D). Additionally, spherical virus particles, approximately 80 nm in diameter with an icosahedral shape, were observed using electron microscopy, consistent with the morphological characteristics of ARV (Figure 1E).

### Genomic characterization

**Figure 2:**
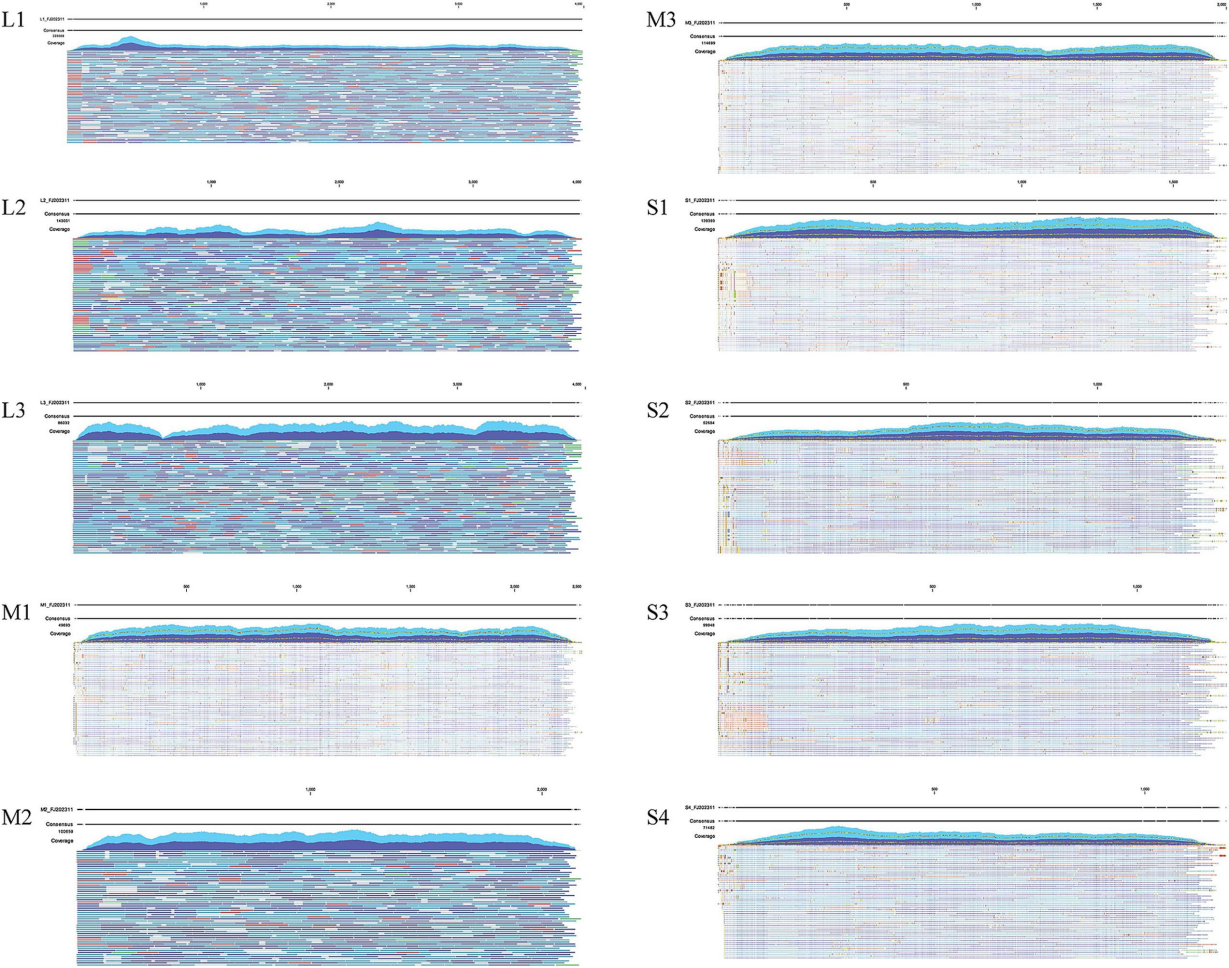
Genome coverage analysis of ARV strain FJ202311. A total of 19,144,330 reads were mapped to the complete genome of FJ202311, yielding an average coverage depth of 81,491 reads.

The CLC Genomics Workbench software was utilized for viral genome assembly. A total of 34,466,524 paired-end sequencing reads, each 100 bases in length, were generated by the MGISEQ sequencer, resulting in 1.45 Gb of clean data (excluding adaptors, mRNA, rRNA, and chicken sequences). The CLC Map Reads to Reference tool was employed to analyze the original sequences, showing that 19,144,330 reads supported the full genome of the FJ202311 strain, with an average coverage depth of 81,491 reads (Figure 2). Additionally, the full genome of the FJ202311 strain was verified through PCR amplification and Sanger sequencing conducted by Sangon (Shanghai, China).

The whole genome sequence of the FJ202311 strain was obtained and deposited in GenBank under accession numbers PQ106581 to PQ106590. As shown in Table 3, the genome of the FJ202311 strain is 23,495 nucleotides (nt) in length, comprising 10 dsRNA segments, which range from 3,959 bp (L1) to 1,192 bp (S4). In the 5’ UTR, the M1 fragment of the FJ202311 strain contains a GCUUUUC motif, while the remaining nine fragments feature a GCUUUUU motif. In the 3’ UTR, four fragments (L2, L3, M1, S2) share an ACUCAUC motif, whereas the remaining six fragments exhibit an AUUCAUC motif.

**Table 3:** Genomic organization of the ARV strain FJ202311.

Genomic segment	Length (bp)	Terminal region sequences (5′-3′)	Size (bp) of the			Viral protein	Protein size (aa)
			5’ UTR	ORF	3’ UTR		
L1	3,959	GCUUUUU…AUUCAUC	21	3,882	56	λA	1,294
L2	3,830	GCUUUUU…ACUCAUC	14	3,780	36	λB	1,260
L3	3,907	GCUUUUU…ACUCAUC	12	3,858	37	λC	1,286
M1	2,283	GCUUUUC…ACUCAUC	12	2,199	72	μA	733
M2	2,158	GCUUUUU…AUUCAUC	29	2031	98	μB	677
M3	1996	GCUUUUU…AUUCAUC	24	1908	64	μNS	636
S1	1,644	GCUUUUU…AUUCAUC	31	291	33	p10	97
				441		p17	147
				981		σC	327
S2	1,324	GCUUUUU…ACUCAUC	15	1,251	58	σA	417
S3	1,202	GCUUUUU…AUUCAUC	30	1,104	68	σB	368
S4	1,192	GCUUUUU…AUUCAUC	23	1,104	65	σNS	368

### Sequence comparison

The nucleotide and amino acid sequences of the FJ202311 isolate were most similar to the PHC-2020-0545 strain in the λC-, μNS-, and σNS-encoding genes (nt: 89.9–92.9%; aa: 94.8–98.6%), the AHZJ19 strain in the λB-encoding gene (nt: 91.9%; aa: 98.7%) and σA-encoding gene (nt: 97.8%; aa: 99.3%), the SDYT2020 strain in the λA-encoding gene (nt: 97.3%; aa: 99.5%), and the K1600657 strain in the σB-encoding gene (nt: 91.5%; aa: 96.7%). Additionally, the FJ202311 isolate was similar to the AHZJ19 strain (nt: 97.7%; aa: 98.5%) and the PHC-2020-0545 strain (nt: 96.5%; aa: 98.6%) in the μA-encoding gene, the 526 strain (nt: 86.3%; aa: 95.7%) and the SD26 strain (nt: 86%; aa: 96.5%) in the μB-encoding gene, the LY383 and SD26 strains (nt: 79.7%) and the K1600657 and 3,211-V-02 strains (aa: 85%) in the p10-encoding gene, and the LY383 and SD26 strains (nt: 71.7%; aa: 72.1%) in the p17-encoding gene.

**Figure 3:**
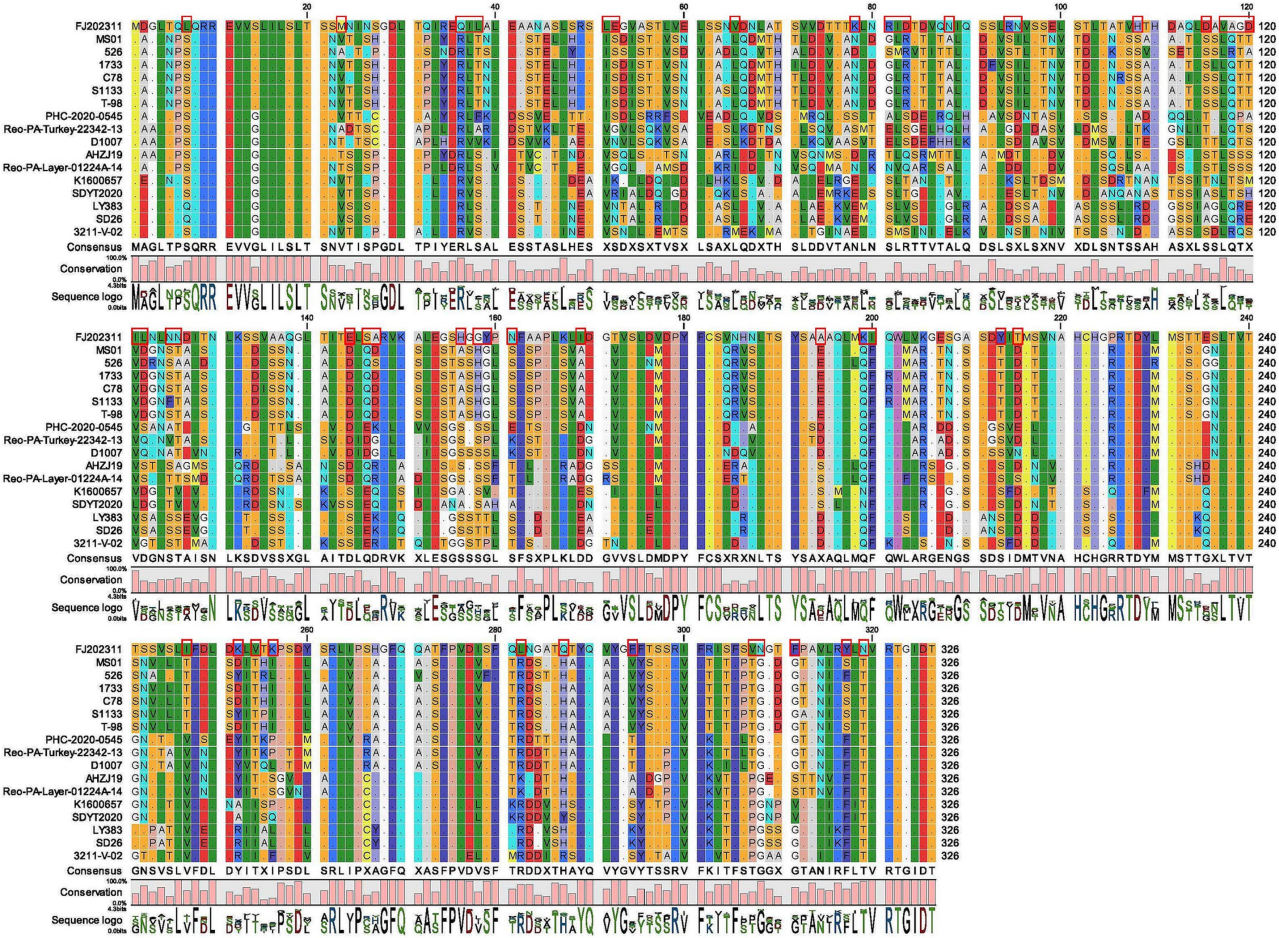
Sequence comparison of the σC amino acid between the FJ202311 strain and 16 other ARV reference strains. The red box highlights amino acids unique to the FJ202311 isolate.

Furthermore, we compared and analyzed the σC amino acid sequences of FJ202311 with the other 16 ARV reference strains. It is notable that the FJ202311 isolate in S1 segment σC-encoding gene has lower identity with the 16 reference strains (nt: 52.5–61.6%; aa:47.1–59.3%). The amino acid alignment of the σC genes showed that 50 amino acids were identified only in the FJ202311 isolate at positions 7 (L), 23 (M), 36 (Q), 37 (I), 38 (L), 51 (L), 52 (E), 65 (V), 78 (K), 81 (R), 82 (I), 83 (D), 88 (N), 94 (R), 95 (N), 108 (H), 115 (D), 117 (V), 118 (A), 119 (G), 120 (D), 121 (I), 122 (L), 125 (N), 126 (N), 144 (E), 146 (S), 147 (A), 156 (H), 158 (G), 159 (Y), 161 (N), 169 (I), 194 (A), 199 (K), 200 (I), 213 (Y), 215 (T), 247 (I), 252 (K), 254 (V), 256 (K), 282 (L), 287 (Q), 294 (F), 307(V), 308 (N), 311 (F), 317 (Y), 319 (N) (Figure 3). The σC proteins were predicted to have molecular weights of 35.2 kDa (the FJ202311 isolate), 34.8 kDa (S1133), 35.1 kDa (PHC-2020-0545), 35.1 kDa (AHZJ19), 35.0 kDa (SDYT2020), 34.8 kDa (LY383) and 35.3 kDa (3211-V-02), and pI of 5.13 (the FJ202311 isolate), 4.76 (S1133), 4.92 (PHC-2020-0545), 4.77 (AHZJ19), 4.69 (SDYT2020), 4.89 (LY383) and 4.82 (3211-V-02). These results indicate slight differences in the size and isoelectric point of σC proteins between the FJ202311 isolate and the 16 reference strains.

### Phylogenetic analysis and visualization analysis

**Figure 4:**
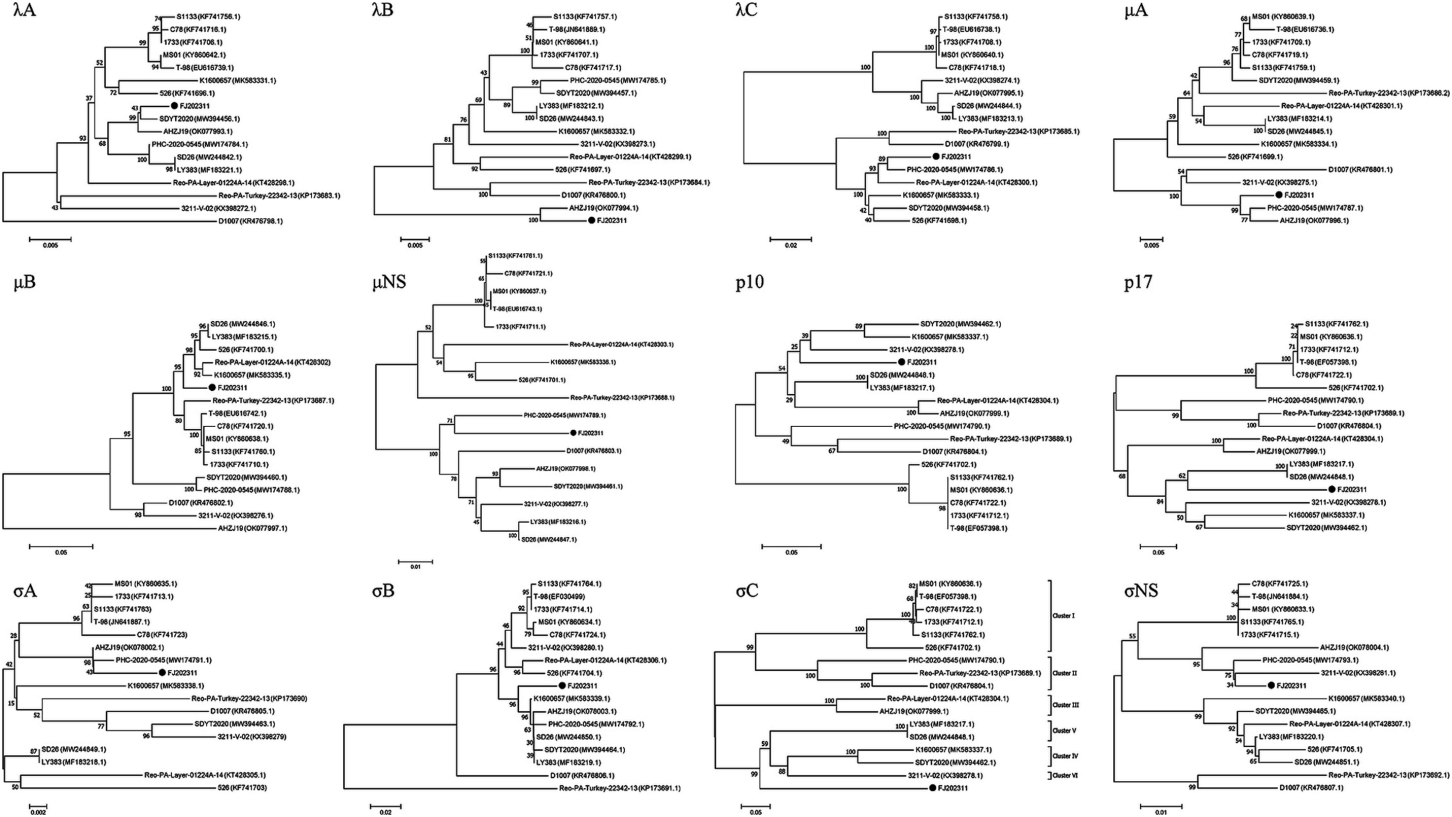
Phylogenetic trees constructed using the neighbor-joining method in MEGA 7.0 based on the 12 proteins (λA, λB, λC, μA, μB, μNS, p10, p17, σA, σB, σC, σNS). The FJ202311 strain is indicated by a black circle.

For further analysis of the genetic evolution of the FJ202311 strain, amino acid sequences of the 12 proteins from each strain were subjected to phylogenetic analyses using MEGA 7 software. As shown in Figure 4, the σC phylogenetic tree revealed that the 16 reference strains clustered into six genotypes, whereas the FJ202311 strain formed a distinct cluster, exhibiting extremely high variability compared to strains in other clusters.

In the λA phylogenetic tree, the FJ202311 isolate and the SDYT2020 strain clustered on the same branch, consistent with sequence comparison results. Similarly, in the λB phylogenetic tree, the FJ202311 isolate and the AHZJ19 strain appeared on the same branch, corroborating the sequence comparison findings. The phylogenetic trees for the λC and μNS proteins showed that the FJ202311 isolate clustered with the PHC-2020-0545 strain, in agreement with the sequence comparisons. For the μA and σA proteins, the phylogenetic analysis demonstrated that the FJ202311 isolate, the PHC-2020-0545 strain, and the AHZJ19 strain clustered on the same branch. In the p17 phylogenetic tree, the FJ202311 isolate was grouped on the same branch with the LY383 and SD26 strains. The σNS phylogenetic tree placed the FJ202311 isolate, the PHC-2020-0545 strain, and the 3,211-V-02 strain on the same branch. In addition, the μB phylogenetic tree grouped the FJ202311 isolate on the same branch as five other strains (LY383, SD26, 526, Reo-PA-Layer-01224A-14, and K1600657). The σB phylogenetic tree placed the FJ202311 isolate with six other strains (LY383, SD26, SDYT2020, PHC-2020-0545, AHZJ19, and K1600657). Similarly, the p10 phylogenetic tree grouped the FJ202311 isolate on the same branch as three strains (SDYT2020, 3,211-V-02, and K1600657).

**Figure 5:**
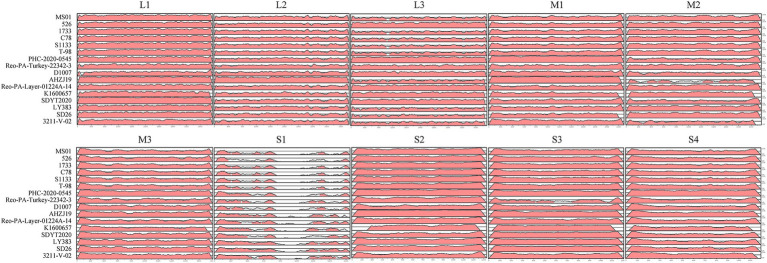
Visualization of the complete genome of the FJ202311 strain and 16 ARV reference strains using the mVISTA online platform. Regions in red denote nucleotide sequence similarities ≥90%, while white regions represent similarities <90%.

For a whole genome comparison of the FJ202311 strain with the other 16 reference strains, full genomic sequences were analyzed using the mVISTA online platform. As depicted in Figure 5, the highest nucleotide sequence similarities were observed in the L2, M1, and S2 segments of the AHZJ19 strain; in the L3, M3, and S4 segments of the PHC-2020-0545 strain; in the L1 segment of the SDYT2020 strain; in the M2 segment of the SD26 strain; and in the S3 segment of the K1600657 strain. Most importantly, the FJ202311 isolate exhibited significantly low sequence identity with the other 16 reference strains in the S1 segment, which encodes the virus attachment protein σC. This suggests that a vaccine formulated with the classical strain may be ineffective against this novel strain.

### Pathogenicity analysis

**Figure 6:**
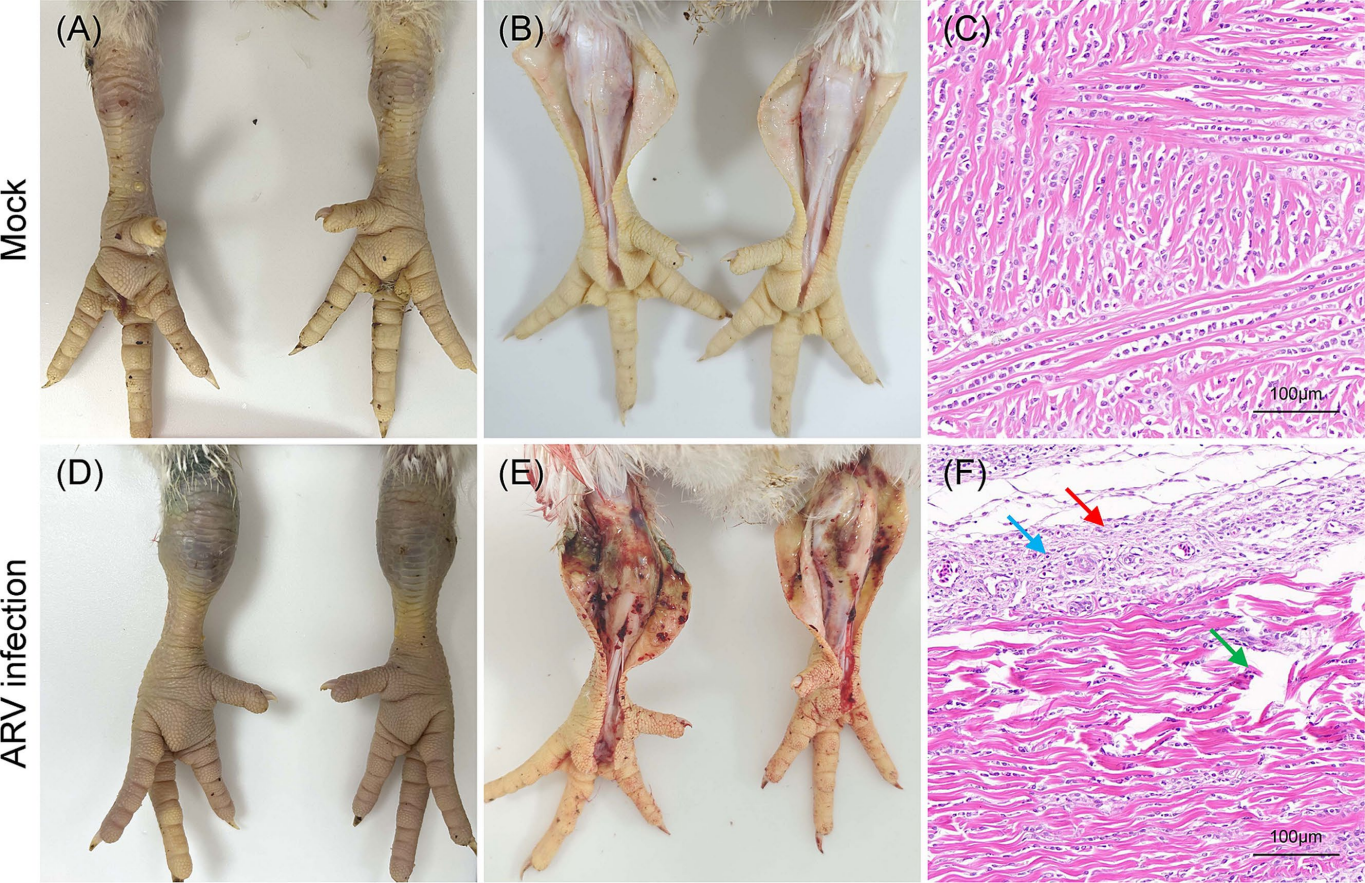
Clinical signs, gross lesions and histological analysis in broilers inoculated with ARV strain FJ202311 at 14 dpi. **(A)** Normal joint appearance in the control group. **(B)** Normal articular cavity appearance in the control group. **(C)** Normal tendon appearance in the control group. **(D)** Swollen joints and bluish-purple discoloration of the tarsal joint in the infected group. **(E)** Severe hemorrhage in the tarsal joint of infected broilers. **(F)** Histopathological analysis showing local collagen fiber disruption (green arrow), significant connective tissue proliferation (black arrow), and focal lymphocyte infiltration (blue arrow) in the tendon.

**Figure 7:**
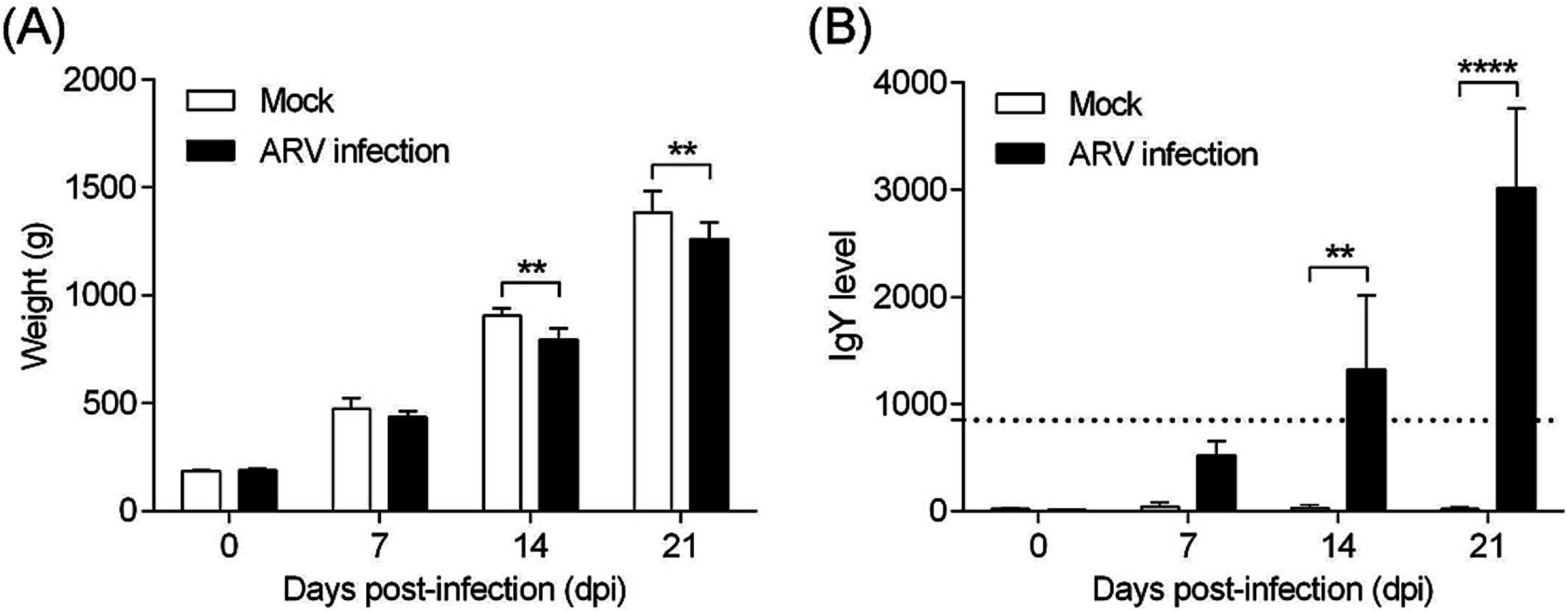
Body weight and ARV-specific antibody levels in broilers. **(A)** Body weight changes in broilers from each group throughout the experiment. **(B)** ARV-specific antibody levels in broilers from each group during the challenge study. Significant differences are indicated by asterisks, **** *p* < 0.0001 and ** *p* < 0.01.

The pathogenicity of the cell-cultured ARV strain FJ202311 (fourth passage), was evaluated in 7-day-old broiler chickens. Throughout the experiment, no clinical signs, gross lesions, or histological abnormalities were observed in the control group (Figures 6A–C). In contrast, chickens in the infected group displayed signs of depression and stunted growth, though no mortality occurred. Swelling of the footpads was first observed at 2 days post-infection (dpi), progressively extended to the tarsal joints by 7 dpi. By 14 and 21 dpi, the tarsal joints were markedly swollen and exhibited a bluish-purple discoloration (Figure 6D). Gross examination of the infected chickens revealed intra-tarsal joint hemorrhages (Figure 6E), while no significant macroscopic lesions were detected in other organs at 14 and 21 dpi. Histopathological analysis showed significant tissue damage localized to the tendons, including disrupted collagen fibers, extensive connective tissue proliferation, focal lymphocytic infiltration, and perivascular cuffing (Figure 6F). These observations indicate a localized inflammatory response in the tendons and surrounding tissues. By 14 and 21 dpi, the mean body weight of infected chickens was significantly lower than that of the control group, with reductions of 11.78 and 8.93%, respectively (*p* < 0.01) (Figure 7A). Additionally, ARV-infected chickens exhibited a strong humoral immune response, with antibody levels significantly increased by 47.73-fold and 154.15-fold at 14 and 21 dpi, respectively, compared to the control group (*p* < 0.01) (Figure 7B).

### Viral distribution and shedding

**Figure 8:**
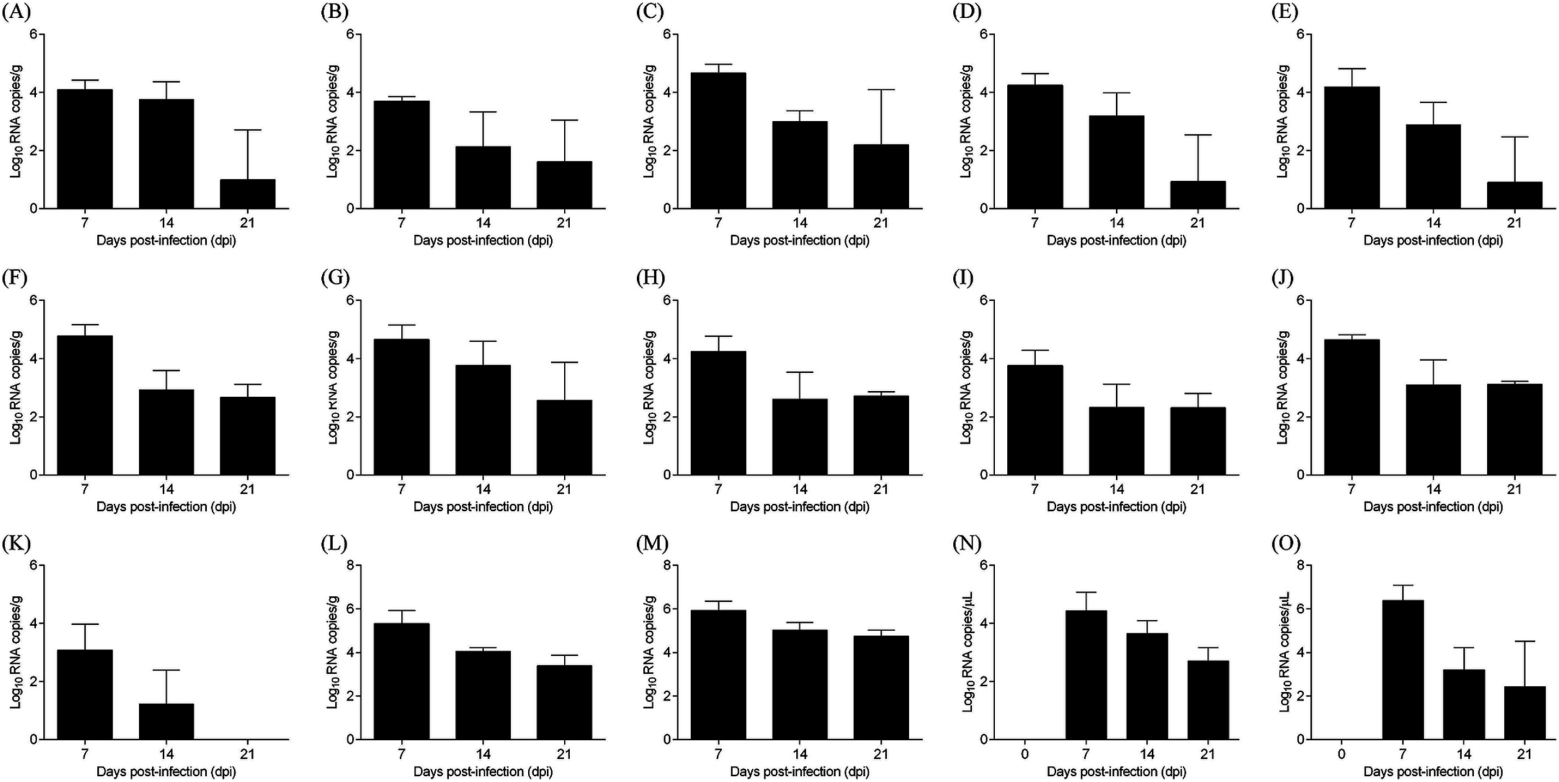
Virus distribution in various samples from ARV-infected chickens. **(A)** Heart, **(B)** Liver, **(C)** Spleen, **(D)** Lung, **(E)** Kidney, **(F)** Bursa of Fabricius, **(G)** Pancreas, **(H)** Small intestine, **(I)** Proventriculus, **(J)** Gizzard, **(K)** Thymus, **(L)** Cecal tonsil, **(M)** Tendon, **(N)** Blood, **(O)** Cloacal swab.

The distribution and shedding patterns of ARV strain FJ202311 were determined using real-time RT-PCR to quantify viral RNA levels in various tissues, blood, and cloacal swabs. As shown in Figure 8, viral loads peaked at 7 dpi, with the highest levels observed in the tendon (10^5.93 copies/g), followed by the cecal tonsils (10^5.32 copies/g). By 14 dpi, viral loads had significantly decreased in most tissues but remained elevated in the tendons (10^5.02 copies/g) and cecal tonsils (10^4.05 copies/g). At 21 dpi, viral RNA persisted at lower levels, with tendons showing the highest residual viral load (10^4.74 copies/g).

Cloacal swabs revealed continuous viral shedding from 7 to 21 dpi, with a peak at 7 dpi (10^6.38 copies/μL), highlighting the potential for environmental transmission. No viral RNA was detected in control chickens. These results demonstrate the broad tissue tropism of FJ202311, with a strong preference for tendon tissues, and provide valuable insights into the strain’s pathogenicity and transmission dynamics.

## Discussion

Reoviruses infect a wide range of hosts, including birds, mammals, and humans, demonstrating remarkable evolutionary adaptability and zoonotic potential (Sellers, 2022; Diller et al., 2023; Song et al., 2008). Their substantial economic impact on the poultry industry, coupled with potential health risks to humans, underscores the necessity for continuous monitoring and characterization of emerging strains (David and Lemay, 2023; Rafique et al., 2024). Since ARV-associated arthritis and tenosynovitis were first described in domestic poultry during the 1950s, numerous strains with varying pathogenicity and host impacts have been reported worldwide, posing significant challenges to epidemic control (Chen et al., 2019; Nour et al., 2023; Rafique et al., 2024). ARVs are primarily transmitted horizontally within flocks via the fecal-oral route, with some strains also capable of vertical transmission (Rafique et al., 2024). In China, ARV infections were first reported in 1985 (Wang et al., 1985), with a marked increase in cases since 2008 (Chen et al., 2019; Yan et al., 2021; Jiang et al., 2023). The dynamic nature and rapid adaptation of ARV strains necessitate ongoing surveillance, development of strain-specific vaccines, and effective management strategies to safeguard poultry health and industry sustainability. In this study, we successfully isolated a novel ARV strain, FJ202311, from a 25-day-old white-feathered Cobb chicken flock in Fujian Province that exhibited clinical signs of arthritis, tenosynovitis, and poor growth. The FJ202311 strain induced severe arthritis and tenosynovitis in broiler chickens, demonstrating its pathogenic potential and significant impact on poultry health. This novel strain represents a valuable resource for virological and serological studies and future vaccine development.

The stable Leghorn male hepatoma (LMH) cell line, commonly used for ARV isolation, was employed to successfully culture the FJ202311 strain. The strain exhibited extensive cytopathic effects (CPE) characteristic of ARV infections, including the formation of large, fused “bloom-like” structures (Lu et al., 2015; Tang et al., 2015). The high viral titer of 10^8.33 TCID_50_/mL reflects the strain’s robust replication capacity and adaptability to *in vitro* conditions. Immunofluorescence and electron microscopy confirmed the isolate as an ARV, displaying the typical icosahedral morphology. These findings are consistent with established ARV isolation methodologies and provide a reliable foundation for further genomic and pathogenicity studies (Yan et al., 2021; Nour et al., 2023).

The complete genome of the FJ202311 strain was obtained by next-generation sequencing and verified by PCR amplification and Sanger sequencing. The whole genome of the FJ202311 isolate was 23,495 nucleotides in length, consist of 10 dsRNA segments ranging from 3,959 bp (L1) to 1,192 bp (S4). Previous studies have shown that the 5’ UTRs and 3’ UTRs of the ARV genome segment are highly conserved, with motifs such as 5’-GCUUUUU-3′ in the 5’ UTRs, and 5′ -UCAUC-3′ in the 3’ UTRs (Su et al., 2006). The FJ202311 strain exhibited these conserved motifs, with minor variations in the M1 segment (GCUUUUC motif). Additionally, the highest nucleotide sequence similarities were observed in the L2, M1, and S2 segments with the AHZJ19 strain; in the L3, M3, and S4 segments with the PHC-2020-0545 strain; in the L1 segment with the SDYT2020 strain; in the M2 segment with the SD26 strain; and in the S3 segment with the K1600657 strain. However, the S1 segment, encoding the σC protein, displayed only 52.5–61.6% nucleotide identity with the 16 reference strains, underscoring its genetic uniqueness.

The σC protein, the most variable ARV protein, mediates viral attachment to host cells, a critical step in infection initiation. It also induces type-specific neutralizing antibodies, playing a pivotal role in host immune response and vaccine development. Its high variability, driven by immune pressure, contributes to viral adaptation and strain diversification, serving as a key marker for ARV classification. Previous studies have categorized ARV strains into six genotypic clusters based on σC sequences (Lu et al., 2015; Ayalew et al., 2017; Palomino-Tapia et al., 2018). For example, Palomino-Tapia et al. (2018) classified ARV strains into Clusters 1–6 in Western Canada during 2012–2017. Similarly, Lu et al. (2015) categorized ARV strains into Clusters 1–6 in Pennsylvania, USA, from 2011–2014. Liu et al. (2023) also divided ARV strains into Clusters 1–6 in China between 2019–2020. In our study, phylogenetic analysis of the σC gene placed FJ202311 in a distinct cluster, separate from the six known genotypic clusters of ARV strains (Lu et al., 2015). The strain exhibited significant amino acid variability (47.1–59.3% identity) compared to reference strains, with 50 unique residues identified in the FJ202311 σC protein. This variability is likely linked to the strain’s ability to adapt to different host environments and may play a role in its pathogenicity. The size and isoelectric point of the σC protein also exhibited slight differences, further emphasizing the strain’s genetic divergence. These findings suggest that FJ202311 represents a novel genotypic lineage, highlighting the ongoing genetic evolution of ARV.

The clinical manifestations of ARV infections are highly variable, with distinct genotypes associated with specific disease symptoms (Rafique et al., 2024). Among these, tenosynovitis and arthritis are the predominant clinical manifestations observed across most ARV genotypes (Rafique et al., 2024). For example, Genotype I is most frequently linked to tenosynovitis and arthritis, where affected birds exhibit swelling and inflammation of the tendons and joints, leading to lameness and impaired mobility. Additionally, this genotype is associated with malabsorption syndrome, characterized by diarrhea, nutrient malabsorption, and poor growth. Respiratory symptoms such as coughing and nasal discharge may also occur, complicating the disease profile (Kant et al., 2003; Hellal et al., 2013; De Carli et al., 2020). Genotype II predominantly causes tenosynovitis and arthritis, similar to Genotype I, but is also closely associated with runting-stunting syndrome. This condition results in uneven growth, underdeveloped chicks, and significant flock performance losses (Kant et al., 2003; De Carli et al., 2020; Kovacs et al., 2022). Genotypes III and V primarily present with tenosynovitis and arthritis, with less frequent reports of other clinical signs. These genotypes cause severe lameness and reduced activity, which adversely impact feed intake and weight gain in affected birds (Kant et al., 2003; Liu et al., 2003; De Carli et al., 2020). Genotype IV exhibits a broader clinical spectrum, combining tenosynovitis, arthritis, runting-stunting syndrome, and malabsorption syndrome. This broader range of symptoms increases the difficulty of diagnosis and control, as affected birds may show signs of multiple overlapping conditions, leading to significant economic losses (Kant et al., 2003; Liu et al., 2003). Genotype VI is characterized exclusively by tenosynovitis and arthritis, which are marked by joint swelling, heat, and pain, causing pronounced lameness. Although restricted to musculoskeletal symptoms, the severity of these signs can result in significant morbidity and mortality in affected flocks (Lu et al., 2015; Egana-Labrin et al., 2019; Zhang et al., 2019). In this study, the novel FJ202311 strain caused severe tenosynovitis and arthritis in broilers, consistent with previous findings on highly pathogenic ARV strains. Clinically, infected broilers exhibited characteristic swelling of the footpads and tarsal joints, progressing to bluish-purple discoloration and significant gross pathology. Histopathological analysis revealed marked collagen fiber disruption, connective tissue proliferation, and focal lymphocytic infiltration in tendon tissues. These findings highlight the localized inflammatory response and structural damage caused by the FJ202311 strain. These lesions resulted in significant growth retardation, with infected broilers exhibiting an 11.78 and 8.93% decrease in mean body weight compared to control broilers at 14 and 21 dpi, respectively.

Interestingly, quantitative RT-PCR analysis revealed significantly higher levels of viral RNA in tendon tissues compared to other organs, confirming that tendons are the primary target organ for ARV infection. This observation aligns with previous studies (Jiang et al., 2021; Yan et al., 2021) and underscores the critical role of tissue tropism in ARV pathogenesis. The pronounced viral load in tendons correlates with the observed clinical and histopathological changes, further emphasizing the diagnostic importance of tendon lesions in ARV-infected birds. Additionally, mutations in the σC protein may enhance the strain’s binding affinity to tendon-specific receptors, contributing to its tissue tropism and pathogenicity. This study provides valuable insights into the FJ202311 strain’s characteristics and pathogenicity. However, several limitations should be acknowledged. First, only broiler chickens were investigated, despite ARV infections being reported across various avian species. This focus on broilers may limit the generalizability of the findings to other poultry or avian species, such as layers and turkeys. Future studies should consider a broader range of species to better understand the host range and pathogenic diversity of ARV strains. Additionally, the molecular mechanisms underlying its pathogenesis remain unclear. Investigating these mechanisms could elucidate host-pathogen interactions and inform the development of more effective vaccines.

In summary, this study successfully isolated and characterized the novel ARV strain FJ202311, which demonstrated considerable genetic variability and induced severe clinical symptoms, including arthritis, tenosynovitis, and impaired growth in infected chickens. These findings not only enhance our understanding of the epidemiological evolution of ARV but also emphasize the critical need for continuous monitoring and genetic analysis of circulating strains. The insights gained from this study regarding the pathogenicity and genetic diversity of ARV are essential for developing more effective control strategies and vaccines. By advancing our knowledge of ARV’s clinical manifestations and transmission dynamics, this research provides a robust foundation for improving disease prevention, surveillance, and management practices. Ultimately, these efforts will contribute to enhanced poultry health and the long-term sustainability of the poultry industry.

## Data Availability

The datasets presented in this study can be found in online repositories. The names of the repository/repositories and accession number(s) can be found in the article/supplementary material.

## Footnotes

^1^ http://genome.lbl.gov/vista/mvista/submit.shtml
